# The effect of group exercise frequency on health related quality of life in institutionalized elderly

**DOI:** 10.11604/pamj.2017.26.35.10518

**Published:** 2017-01-24

**Authors:** Nivash Rugbeer, Serela Ramklass, Andrew Mckune, Johan van Heerden

**Affiliations:** 1 Biokinetics, Exercise and Leisure Sciences (Sport Science), School of Health Sciences, UKZN, Westville Campus, Durban, South Africa; 2 School of Clinical Medicine, College of Health Science, Medical Campus, University of Kwa-Zulu Natal, Main Building, Durban, South Africa; 3 University of Canberra Research Institute for Sport and Exercise (UCRISE), Canberra, ACT, 2601, Australia

**Keywords:** Health related quality of life, mental health, exercise programme, aging, institutionalization, social health, exercise frequency

## Abstract

**Introduction:**

The study aimed to determine the effect of group exercise frequency on health related quality of life in institutionalized elderly.

**Methods:**

One hundred participants were recruited for voluntary participation from five aged care facilities, with inclusion being based on the outcome of a medical assessment by a sports physician. A quasi-experimental design was used to compare the effect of a 12 week group exercise programme on two groups of participants using pre-test and post-test procedures.

**Results:**

A significant difference was noted in social function post training 2X/week (MD = -13.85, 95% CI [-24.66, -3.38], p = 0.017, d = 0.674) and 3X/week (MD = -13.30, 95% CI [-21.81, -5.59], p = 0.003, *d* = 0.712) a week. Training 3X/week a week provided an additional benefit in vitality (MD = -7.55, 95% CI [-13.16, -1.91], p = 0.018, *d* =0. 379). Improvements in mental component summary scale post training 2X/week (MD = -4.08, 95% CI [-7.67, -0.42], p = 0.033, d = 0.425) and 3X/week (MD = -6.67, 95% CI [-10.92, -2.33], p = 0.005, *d* = 0.567) a week was further noted.

**Conclusion:**

Mental health and social health benefits can be obtained irrespective of exercise frequency 2X/week or 3X/week. The exercise intervention at a frequency 3X/ week was more effective in improving mental component summary due to a larger effect size obtained compared to the exercise frequency of 2X/week. Additional benefits in vitality were achieved by exercising 3X/week. This may assist the elderly in preserving their independence.

## Introduction

Aging is a complex and inevitable process, which leads to a decline in the body’s physiological system and physical capacity [1]. The process of aging may increase the occurrence of chronic diseases and conditions such as hypertension, cardiovascular disease, diabetes, cancer and osteoporosis [2]. Aging is commonly characterized by a progressive and general impairment of function, resulting in vulnerability to environmental challenges, and a growing risk of disease and disability [3]. To combat the challenges experienced due to aging, the World Health Organization initiated the ‘Active Aging’ policy to reduce inactivity and improve health related quality of life. “Active aging is the process of optimizing opportunities for health, participation and security in the elderly, to enhance quality of life as people age” [4].

There is compelling scientific evidence worldwide, which suggests that a structured exercise programme can improve the physiological functioning, health related quality of life and functional ability of older persons [1, 5–7]. Despite the benefits of structured exercise, many older persons lead sedentary lifestyles [8]. Persons over the age of 55 years have the lowest reported moderate to vigorous physical activity levels globally, with an increase in age being associated with increased inactivity [8].

In an urban South African study, 49.7% of elderly persons did not meet the minimum guideline of 150 min of physical activity a week [8]. Inactivity is associated with increased risk of heart disease, type 2 diabetes, hypertension and osteoporosis [9, 10].

Health related quality of life (HRQoL) is a state of wellbeing or happiness experienced by an individual despite the presence of illness or disability [11]. In the elderly, it is best described in relation to functional status, independence and the ability to perform activities of daily living efficiently [12]. The Medical Outcomes Study 36-Item Short-Form Health Survey (SF-36) is a widespread, reliable and valid instrument that is used to measure HRQoL in the elderly [11]. It consists of eight subscales namely: role physical (RP), bodily pain (BP), general health (GH), physical functioning (PF) vitality (VT), role emotional (RE), social functioning (SF), and mental health (MH). The scores range from 0 to 100 for each subscale, with higher scores closer to 100 indicating a better HRQoL [13, 14]. The eight subscales are encapsulated into physical component summary (PCS) and mental component summary (MCS) respectively [15].

A lack of physical stimulation results in functional and health disorders, which negatively affects HRQoL [16], while physical activity promotes independence by improving functional capacity and physical health. A systematic review assessing physical activity and quality of life, found that, physical activity had a positive effect on physical [17], psychological [18] and emotional wellbeing [19]. Physical activity is reported to improve vitality, mental and psychological health, and to support moderate improvements in emotional, physical, overall health, social relationships and pain [7].

A number of cross sectional studies concluded that moderate or high intensity exercises were associated with improvements in the following scales of pain [20, 21], physical [22], vitality [20, 21, 23], mental health [20] and general health [24]. Another study conducted in Japan, concluded that HRQoL was associated moderate physical activity in elderly men [25]. A frequency of at least five times a week was associated with better social and physical domains of HRQoL [22].

A systematic review postulated, to achieve benefits in health indicators and quality of life, a multicomponent exercise program is preferred, and should consist of aerobic, muscular endurance, stability/balance and flexibility exercises [5]. Most studies in the elderly implemented exercise programmes with a frequency of two and three times a week, ranging in duration from 3-12 months [5]. In the community dwelling elderly a multicomponent exercise programme resulted in significant improvements in mobility and balance [26]. A high frequency intervention (≤3X/week) in institutionalized elderly resulted in fewer hospital visits and lower risk of mortality [27]. Moderate frequency (2X/week) interventions had a desirable effect on balance in “pre-frail” elderly, substantially reducing the risk of falling at 1-year follow-up [28].

The inability to perform activities of daily living can be a major problem for people living in aged care facilities, either due to a loss of functioning or independence [29, 30]. An international study showed that those who are physically active have a better quality of life and mental health than those with sedentary lifestyles [31]. A lack of independence and inactivity predisposes such persons to chronic disease, particularly those living in aged care facilities. While the above finding is well documented internationally in community dwelling elderly, little research has been conducted in institutionalized homes globally and in the South African context. Therefore, the study aimed to investigate the effect of group exercises 2X/week and 3X/week on HRQoL ininstitutionalized elderly.

## Methods

### Subjects

The study population comprised of individuals who were 60 years of age and older residing in an aged care facility within a 30 kilometre radius of the Durban central business district (CBD). A listing of all government supported elderly care facilities located within a 20 km radius of the Durban CBD was obtained from the Department of Social Development, from which five elderly care homes were randomly selected. All residents who were interested in participating in the study were invited to be assessed to establish whether they met the inclusion criteria. The outcome of a physical assessment conducted by a sports medicine physician, determined, participation in the intervention. From the eligible participants, 20 were randomly selected in each aged care facility, with a total of 100 for the study. They were randomly all allocated numbers from 1 to 20, and the fish bowl technique was used to identify ten participants for Group A (all odd numbers) and ten for Group B (all even numbers). Group B exercised two times a week and group A exercise three times a week for 12 weeks. Individuals were excluded if they were < 60 years of age, had undergone hormone supplementation, were unable to participate based on a medical assessment and were participating in other research/clinical trials. The study was designed as a three-month (12 week) intervention, exercise frequency was three times a week, two sessions per week was introduced as a control arm into the quasi-experimental design, and to establish any difference in effect between the two groups. Participants had to attend 80 % of the exercise sessions. However, as a result of hospital visits and illness during the exercise intervention, 83 participants completed the study (Group A = 47 and Group B = 37)

### Study design and procedure

**Figure 1:**
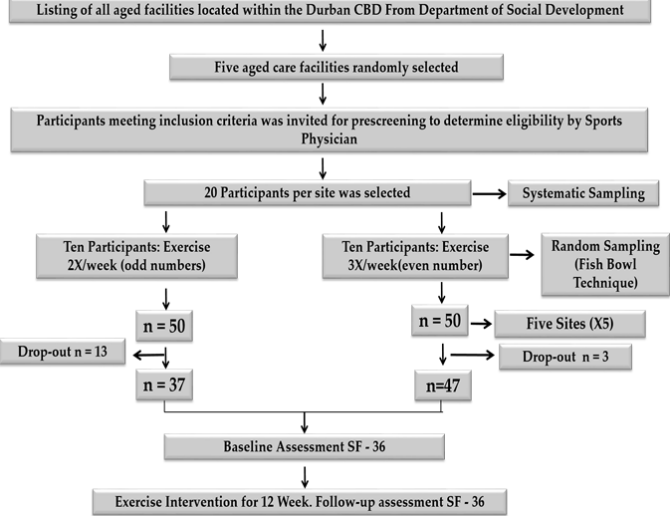
Outline of the study

A quasi-experimental (Figure 1) design was used to compare the effect of a 12 week group exercise programme on two groups of participants using pre-test and post-test procedures. Ethical clearance for this study was obtained from the University of KwaZulu-Natal, School of Health Sciences Research Committee and from the UKZN Biomedical Research Ethics Committee (BE251/11). Permission to conduct the study was granted by the Department of Social Development and each of the five participating aged care facilities. Participation in the study was voluntary and participants could withdraw from the study at any point in time. A pilot study of the SF-36 survey was conducted by students with an exercise background from the College of Health Sciences and a trained research assistant. This was done to evaluate the feasibility and reliability of the SF-36 at one of the five aged care facilities in five voluntary participants who met the inclusion criteria.

At the start of the 12 week study, a baseline assessment using the SF-36 survey was conducted. Follow-up assessments were conducted post intervention. The exercise intervention was adapted from American College of Sport Medicine (ACSM). The exercise programme was conducted 3X/week for Group A (Monday, Wednesday and Friday) and 2X/week for Group B (Monday & Friday) at the five sites. The total duration of exercise increased from 50 to 80 minutes per session. Sessions were conducted each morning between 08h00 and 10h00, at least 60 minutes after breakfast. Each class consisted of a 10 minute warm up, followed by 45 minutes of strength, endurance and mobility/balance exercises, and concluded with a five-minute cool down and stretching routine.

The warm-up included progressive exercises that involve dynamic stretching, continuous rhythmic endurance activities such as easy walking, light marching, toe and heel presses and low knee lifts. The warm-up included rehearsal (step by step but slower tempo) of exercise sequences, as well as specific joint mobility exercises (e.g. arms overhead and circles along with low intensity endurance exercise). Intensity was monitored using Borg’s Rating of Perceived Exertion (RPE) 6 – 20 point scale [32]. Accordingly, the RPE for this population was maintained between 9 and 10.

Endurance training involved walking, which required using the larger muscle groups, and requires rhythmic and continuous movement. Intensity for the first 3 weeks was equivalent to 10 to 11 on the RPE scale (light), while during weeks 4 to 9 the intensity was increased to 12 to 13 on the RPE scale (somewhat hard) and maintained for weeks 10 to 12. However, the duration of exercise was increased over the 12 week exercise programme from three bouts of 5 minutes (week 1 to 3), to two bouts of 10 minutes (weeks 4 to 9), and finally two bouts of 15 minutes (weeks 10 to 12).

The study incorporated resistance exercises for developing muscle endurance, strength and power. The following 10 exercises are deemed appropriate for the elderly and was used to train the entire body: Leg press or squat; Knee extension; Knee curl; Calf raise; Chest press; Seated row; Upright row; Arm curl; Shoulder press; Abdominal/core exercise. Ten repetitions per set of exercise were performed over the 12 week program. The number of sets increased from one in the first 4 weeks to two sets during weeks 5 to 8, and to three sets from weeks 9-12. Abdominal strengthening exercises were used to develop the core and abdominal muscles.

After each training session static flexibility and relaxation activities at a low intensity were performed to allow the body to adjust from exertion to rest. A stretch was applied twice to each muscle group of the body, while relaxation strategies (slow deep breathing) were encouraged between stretches and at the end of activity. Each stretch was held to a point of gentle tension but not pain, for a period of 15-30 seconds.

the data were analyzed using the Statistical Package for Social Science Version 18.0 (SPSS) for Windows software. Descriptive (means and standard deviations) and parametric (paired t-tests) statistics were used to test the variance among the groups for the subscales and component summaries. Data were bootstrapped to minimize bias and confounding variables. Effect sizes (d) was calculated to determine practical significance, as recommended by Cohen (1988), who proposed that an ES of 0.2 represents a small effect, 0.5 a medium effect and 0.8 a large effect. A p value of <0.05 was considered statistically significant [33].

## Results

The study consisted of 79% females and 21% males, with a mean age of 73 years (SD 7.57) (Table 1). A large percentage of the participants in the study were of Indian ethnicity (72%). Over half of the participants were widowed (53%) and had a mean body mass index of 28.07 kg/m^2^.

**Table 1:** Demographic profile of the elderly (N and % =100)

Demographics		N	Demographics		N
**Gender**	Males	21	**Age**	60-69	37
	Females	79		70-79	42
**Marital Status**	Married	15		80-89	21
	Widowed	53	**Racial Group**	Indian	72
	Never married	19		Blacks	1
	Divorced	13		Coloured	11
				White	16

The mean age of participants who exercised twice and thrice a week were 71 and 72 years respectively (Table 2). A similar baseline profile was noted for both groups, with the majority of participants female and Indian (Table 2).

**Table 2:** Demographic profile of the elderly, who, exercised 2X/week and 3X/week

Demographic variable	Group that Exercises 2X / week n (%)	Group that Exercises 3X / week n (%)
Age (mean)	71 years	72 years
**Race**		
Indians	31 (84)	33 (70)
African	1 (3)	0 (0)
Coloured	2(5)	4 (9)
Whites	3 (8)	10 (21)
**Gender**		
Males	8 (22)	10 (21)
Females	29 (78)	37 (79)
**Marital Status**		
Married	9 (24)	3 (6)
Widowed	18 (49)	26 (55)
Never Married	6 (16)	10 (21)
Divorced	4 (11)	8 (17)

Pre-training mean scores of social functioning of elderly participants who exercised 2X/week 82.43 (SD = ±26.75) and post training 2X/week was 96.28 (SD = ±11.36). A paired t-test revealed that this difference was statistically significant, t (36) = -2.69, p =0.017, the mean difference (MD = -13.85, 95% CI [-24.66, -3.38], p = 0.017), demonstrating a medium effect size, *d* =0.674 (Table 3).

**Table 3:** Effect of participation in a group exercise program 3X/week (for 12 weeks) on SF-36 subscales comparing pre and post training

Subscales			Exercised 3X/Week (n=47)					
	Pre-TestM (SD)	Lower CI	Upper CI	Post-TestM (SD)	Lower CI	Upper CI	ES	P
PF	81.06 (±16.32)	76.06	85.64	85.00 (±17.63)	79.85	89.79	0.232	0.112
RP	90.43 (±25.83)	82.45	97.34	86.17 (±27.49)	78.72	92.55	-0.160	0.472
BP	78.67 (±23.81)	72.02	85.04	73.35 (±24.74)	66.43	80.21	-0.219	0.232
GH	83.62 (±13.94)	79.73	87.77	82.55 (±16.87)	78.09	86.70	-0.069	0.731
VT	63.62 (±19.94)	57.77	69.26	71.17 (±19.95)	65.85	76.92	0.379	**^+^0.018**
SF	82.71 (±23.54)	76.06	89.10	96.01 (±11.97)	92.82	98.67	0.712	**^+^0.003**
RE	95.04 (±20.83)	87.23	100.00	95.74 (±13.22)	91.50	99.29	0.040	0.850
MH	82.21 (±15.22)	77.79	86.64	87.32 (15.23)	82.64	91.25	0.336	**^+^0.026**

Abbreviation: PF = physical functioning, RP = role physical, BP = bodily pain, GH = general health, VT = Vitality, SF = social functioning, RE = role emotional, MH = Mental Health, M= mean scores, SD = standard deviation, M = mean scores, SD = standard deviation, ES = effect size: small ≥ 0.1, medium ≥ 0.2, and large ≥ 0.5, BCa 95% CI = bias corrected and accelerated bootstrap confidence intervals * Significant at *P*<0.05.

The participants mean vitality score pre exercise in the group that exercised 3X/week was 63.62 (SD = ±19.94) compared with post training 3X/week that was 71.17 (SD = ±19.95) (Table 3). This difference was statistically significant, t (46) = -2.73, p =0.018, the mean difference (-7.55, 95% CI [-13.16, -1.91]) demonstrating a small effect size, *d*= 0.379 (Table 4).

**Table 4:** Effect of participation in a group exercise program 3X/week (for 12 weeks) on SF-36 subscales comparing Pre and Post training (paired t - test)

Exercised 3X / Week (n = 47)							
Subscales	Pre-Test M (SD)	Post-Test M (SD)	Mean Difference	Mean Difference (Lower 95%CI)	Mean Difference (Upper 95%CI)	ES	P
PF	81.06 (16.32)	85.00 (17.63)	-3.94	-8.62	1.02	0.232	0.112
RP	90.43 (25.83)	86.17 (27.49)	4.26	-5.86	15.20	-0.160	0.472
BP	78.67 (23.81)	73.35 (24.74)	5.32	-3.23	13.46	-0.219	0.232
GH	83.62 (13.94)	82.55 (16.87)	1.06	-4.40	6.81	-0.069	0.731
VT	63.62 (19.94)	71.17 (19.95)	-7.55	-13.16	-1.91	0.379	**0.018**
SF	82.71 (23.54)	96.01 (11.97)	-13.30	-21.81	-5.59	0.712	**0.003**
RE	95.04 (20.83)	95.74 (13.22)	-0.71	-9.79	6.38	0.040	0.850
MH	82.21 (15.22)	87.32 (15.23)	-5.12	-9.30	-0.26	0.336	**0.026**

Abbreviation: PF = physical functioning, RP = role physical, BP = bodily pain, GH = general health, VT = Vitality, SF = social functioning, RE = role emotional, MH = Mental Health, M= mean scores, SD = standard deviation, M = mean scores, SD = standard deviation, d = effect size: small ≥ 0.1, medium ≥ 0.2, and large ≥ 0.5, BCa 95% CI = bias corrected and accelerated bootstrap confidence intervals. Significant at *P*< 0.05.

The elderly’s mental health mean score pre exercise 3X/week was 82.21 (SD = ±15.22) compared with post training 3X/week that was 87.32 (SD = ±15.23). This difference was statistically significant, t (46) = -2.29, p = 0.026, the mean difference (-5.12, 95% CI [-9.30, -0.26]), demonstrating a small effect size, *d*= 0.336 (Table 4).

Pre-training mean scores of social functioning of the elderly participants that exercised 3X/week was 82.71 (SD = ±23.54) compared with post training 3X/week that was 96.01 (SD = ±11.97). This difference was statistically significant, t (46) = -5.56, p = 0.003, the mean difference (-13.30, 95% CI [-21.81, -5.59]), demonstrating a medium effect size, *d*= 0.712 (Table 4).

Promising trend was noted among physical functioning (MPD = -3.94,95% CI [-8.62, 1.02], t (46) = -1.64, p = 0.112) demonstrating a small effect size, *d* = 0.232 (Table 4)

**Figure 2:**
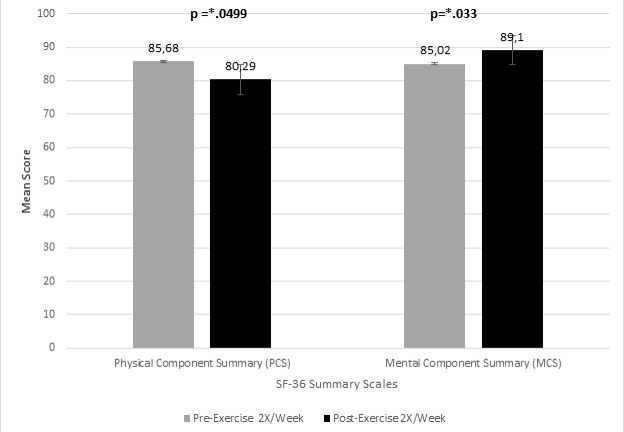
Effect of participation in a group exercise program 2X/week (for 12 weeks) on SF-36 physical and mental component summary comparing pre and post training(paired t - test)

The participants physical component summary scale mean score pre-exercise 2X/week was 85.68 (SD = ±11.22, 95% CI [81.94, 89.31]) compared with post training 2X/week that was 80.29 (SD = ±17.14, 95% CI [73.98, 85.24]). This difference was statistically significant, t (36) = 2.09, p =0.050, the mean difference (5.39, 95% CI [0.70, 10.77]), demonstrating a medium effect size, *d*=-0.302. (Figure 2). The elderly’s mean mental component summary scale pre exercise 2X/week was 85.02 (SD = ±10.04, 95% CI [81.54, 88.10]) compared with post training 2X/week that was 89.10 (SD = ±7.90, 95% CI [86.47, 91.38]). This difference was statistically significant, t (35) = -2.36, p = 0.033, the mean difference (-4.08, 95% CI [-7.67, -0.42]), demonstrating a small effect size, *d*= 0.452. (Figure 2).

**Figure 3:**
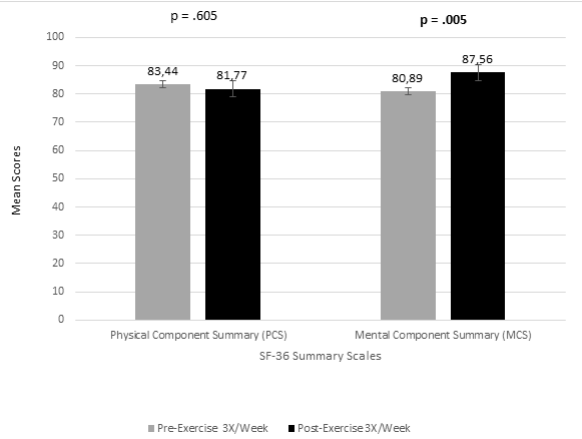
Effect of participation in a group exercise program 3X/week (for 12 weeks) on SF-36 physical and mental component summary comparing pre and post training(paired t - test)

Pre-training mean scores of mental component summary scale of the elderly “participants that exercised 3X/week was 80.89 (SD = ±13.55, 95% CI [76.82, 84.77]) compared with post training 3X/week that was 87.56 (SD = ±9.63, 95% CI [86.47, 90.31]). This difference was statistically significant, t (46) = -3.29, p = 0.005. The mean difference (-6.67, 95% CI [-10.92, -2.33]), demonstrated a medium effect size, *d* =0.567 (Figure 3).

## Discussion

There was a significant difference in social functioning, vitality and mental health comparing pre and post training thrice a week, while social functioning improved irrespective of the frequency of exercise. Exercising twice a week resulted in an improvement in general health post exercise. It could be postulated that in general there was a direct relationship between frequency of exercise and HRQoL. Frequency of participation in physical activity at least five times a week (150 minutes of physical activity per week) was associated with better domains of quality of life, namely physical and social domains conducted in the United Kingdom [22]. Similar findings were noted in the current study, indicating favorable outcomes of social functioning with more frequent structured exercise. The exercise intervention, irrespective of frequency, twice or thrice a week, was effective in improving social functioning.

A similar trend was noted in a study that reported moderate accumulation of physical activity being an important determinant of HRQoL in the older Japanese men [25]. Clinically, greater exercise frequency per week may have a direct relationship between health related quality of life and elderly residing in aged care facilities.

Exercise has recently been found to preserve the functioning of the aging brain [34] and increases brain derived neurotrophic factors in the hippocampus [35]. Brain derived neurotrophic factors may be an important mediators in reducing cognitive decline, which effects a persons’ autonomy [36]. an international study reported that supervised group exercise twice a week for 45 minutes improved the mental HRQoL [37]. In the current study, the frequency of the exercise (2X/week and 3X/week) resulted in elevated mental component summary, hence, group exercise had a desirable effect on mental health in the elderly residing in long-term care facilities.

There was a significant difference in mental health in the group that exercised 3X/week. A significant difference was observed in the mental component summary comparing pre and post training twice a week and thrice a week. Clinically, the exercise intervention at a frequency of thrice a week was effective in improving mental component summary due to a medium effect size obtained compared to the exercise frequency of twice a week. The findings are similar to a study conducted among institutionalized elderly residing in Malaysia, which indicated that a multi-component exercise programme significantly improved the mental component and physical component summaries [38]. Their results are similar to the current study with respect to mental but not physical component summary. This could be attributed to different contextual factors, cultural attitudes to aging, and lack of frequency of exercise to elicit benefits in the physical domain of HRQoL [39]. It is quite clear from the study that frequency of exercise required to obtain mental and physical health benefits vary in the context of institutionalized elderly. Further targeted longitudinal intervention based studies are required to investigate the impact of intensity, frequency, duration and type of physical activity that is required to obtain mental and physical health benefits.

## Conclusion

The purpose of the study was to determine the effect of group exercise frequency on HRQoL of the elderly living in institutionalized care facilities. Exercise frequencies of 2X/week and 3X/week had a desirable effect on mental health and social functioning in the elderly residing in long-term care facilities. Overall mental health and social functioning benefits can be obtained irrespective of exercise frequency 2X/week or 3X/week. The exercise intervention at a frequency of 3X/week was more effective in improving mental component summary due to a larger effect size obtained compared to the exercise frequency of twice a week. Additional benefits in vitality were achieved by exercising thrice a week. The findings suggest that group exercise is an effective intervention for improving and preserving mental HRQoL [40]. This may assist the elderly in preserving their independence and accomplishing activities of daily living safely and effectively.

### What is known about this topic

- Exercise improves HRQoL, balance and functional ability in community dwelling older persons, however the there is a paucity in literature about the benefits in institutionalized elderly;
- Exercise improves mental health in community dwelling older persons;
- Exercise improves physical health in community dwelling older persons.

### What this study adds

- Overall mental health and social functioning benefits can be obtained irrespective of exercise frequency 2X/week or 3X/week;
- The exercise intervention at a frequency of 3X/week was more effective in improving mental component summary due to a larger effect size obtained compared to the exercise frequency of twice a week;
- Additional benefits in vitality were achieved by exercising 3X/ week.
